# Aesthetic Emotions and Aesthetic People: Openness Predicts Sensitivity to Novelty in the Experiences of Interest and Pleasure

**DOI:** 10.3389/fpsyg.2015.01877

**Published:** 2015-12-09

**Authors:** Kirill Fayn, Carolyn MacCann, Niko Tiliopoulos, Paul J. Silvia

**Affiliations:** ^1^School of Psychology, University of Sydney, SydneyNSW, Australia; ^2^Department of Psychology, University of North Carolina at Greensboro, GreensboroNC, USA

**Keywords:** Openness/Intellect, interest, knowledge emotions, aesthetics, personality processes, appraisals, multi-level modeling

## Abstract

There is a stable relationship between the Openness/Intellect domain of personality and aesthetic engagement. However, neither of these are simple constructs and while the relationship exists, process based evidence explaining the relationship is still lacking. This research sought to clarify the relationship by evaluating the influence of the Openness and Intellect aspects on several different aesthetic emotions. Two studies looked at the between- and within-person differences in arousal and the emotions of interest, pleasure and confusion in response to visual art. The results suggest that Openness, as opposed to Intellect, was predictive of greater arousal, interest and pleasure, while both aspects explained less confusion. Differences in Openness were associated with within-person emotion appraisal contingencies, particularly greater novelty-interest and novelty-pleasure relationships. Those higher in Openness were particularly influenced by novelty in artworks. For pleasure this relationship suggested a different qualitative structure of appraisals. The appraisal of novelty is part of the experience of pleasure for those high in Openness, but not those low in Openness. This research supports the utility of studying Openness and Intellect as separate aspects of the broad domain and clarifies the relationship between Openness and aesthetic states in terms of within-person appraisal processes.

## Introduction

“*It is art that makes life, makes interest, makes importance... and I know of no substitute whatever for the force and beauty of its process.”*

-Henry James

Making and appreciating art is a quintessentially human behavior, but not everyone would agree with the sentiment expressed by Henry James above. Divergent opinions about the importance of art and experiences with art make the study of individual differences a crucial part of aesthetic science—after all, it is said that beauty is in the eye of the beholder. However, in psychological aesthetics there are still gaps in what is known about both the beauty and the beholder. Psychological aesthetics has primarily focused on one aspect of the aesthetic experience in the form of liking, pleasure and preference. Aesthetics associations with personality—primarily Openness/Intellect—have focused almost exclusively on individual differences in liking different types of art. Further, little work has gone into understanding the processes underlying the relationship between aesthetics and Openness/Intellect. This is problematic because the nature of the personality/art appreciation relationship could seem circular, given that personality items directly mention aesthetic engagement when measuring Openness/Intellect.

In the current study, we extend previous research investigating the relationship between Openness/Intellect and aesthetic appreciation in three ways. First, we model the appraisal processes underlying the emotions of interest, pleasure, and confusion. This extends previous research by considering three distinct emotions rather than pleasure only. Second, we test whether the aspects of Openness and Intellect differentially predict these three emotional states. This extends previous research by considering the two different aspects of Openness/Intellect, rather than the broad domain only. Third, we test whether the aspects of Openness and Intellect differentially predict within-person appraisal processes underlying these three emotional states. This extends previous research by considering within-person processes, rather than between-person associations only. By integrating these various elements we intended to answer the question: *Why* are those higher in Openness/Intellect more aesthetically engaged?

### Aesthetic People

Openness/Intellect is the personality domain of the aesthetically sensitive, according to many areas of research. It is the best predictor of positive aesthetic attitudes and participation in aesthetic activities such as visiting museums, reading literature, and creating art (McManus and Furnham, 2006). Previous findings have demonstrated Openness/Intellect to be the best personality predictor of artistic creativity (Feist, 1998; Silvia et al., 2009b) and vocational interests related to the arts (Barrick et al., 2003). Most importantly, Openness/Intellect is a consistent predictor of aesthetic appreciation, which has been shown to be highly variable (Vessel and Rubin, 2010). Several studies indicate that Openness/Intellect is associated with liking a broad range of artistic types including abstract, representational, pop, renaissance, cubism, Japanese, and unpleasant art (Furnham and Walker, 2001; Rawlings, 2003; Chamorro-Premuzic et al., 2009, 2010). Openness/Intellect therefore is a domain of personality that explains individual differences in creating, seeking, and appreciating art.

Openness/Intellect is an unusually heterogeneous personality domain, and recent work suggests that it can be represented with two major aspects: *Openness* and *Intellect* (DeYoung et al., 2007, 2012; Woo et al., 2014). Johnson (1994) poetically described Openness as interest in beauty and Intellect as interest in truth, suggesting that they are both information-seeking traits diverging in the types of situations that elicit interest.

Intellect is associated with fluid and crystallized intelligence and with scientific creativity, while Openness is associated with artistic creativity, implicit learning ability, and crystallized intelligence (Kaufman et al., 2010; Nusbaum and Silvia, 2011; Kaufman, 2013). DeYoung (2014) distinguishes the aspects on the basis of different styles of cognitive exploration, with Openness reflecting individual differences in exploration through perceptual or sensory information, and Intellect reflecting individual differences in learning and exploration of abstract information. Johnson’s (1994) and DeYoung’s (2014) distinctions suggest that Openness, as opposed to Intellect, is the aspect primarily associated with appreciation of visual art. Further distinctions based on emotional experiences have also emerged. Silvia and Nusbaum (2011) showed that Openness, and not Intellect, is associated with unusual aesthetic experiences such as chills, feeling touched, and absorption, suggesting differences between the aspects in the propensity to experience states that have been linked to broad definitions of aesthetic experiences. Given the distinction between Openness and Intellect we aimed to test their differential roles in aesthetic experiences.

### Aesthetic Emotions

Nearly all research on the link between personality and aesthetic appreciation, like aesthetics research more generally, has focused on how much participants liked or disliked an artwork (e.g., Furnham and Walker, 2001; Rawlings, 2003; Chamorro-Premuzic et al., 2009). Since the pioneering work of Berlyne (1971), most models of aesthetics concern themselves with states of pleasure, liking, or preference. Silvia (2009) argued that, while important, such evaluations do not take into account the breadth of emotions felt in response to art. A similar trend exists within the research in the emerging field of *neuroaesthetics*, which has almost exclusively focused on the evaluation of something as pleasing or beautiful (Fayn and Silvia, 2015). Such a reductionist approach runs the risk of missing meaningful individual differences in aesthetic experiences and in understanding the ways in which personality traits manifest in such experiences. Emotions felt in response to aesthetic objects—categorized within this paper as aesthetic emotions—are varied and include interest, confusion, pleasure, anger, and even disgust (Silvia, 2012). The term aesthetic emotions is not used to suggest a separate group of emotions only felt in response to aesthetic objects. Rather, it is used to group the states that have been observed to occur in response to aesthetic objects.

The distinction between liking versus disliking something may be a valid indicator of pleasure, but it does not represent the depth and complexity of aesthetic emotions. A group of emotions frequently felt in response to art, yet distinct from pleasure, are the *knowledge emotions*. The knowledge emotions—interest, awe, beauty, confusion, and surprise—associated with beliefs about thoughts and knowledge, they stem from epistemic goals, and arise from metacognitive processes (Silvia, 2010, 2012). Several emotional states may fit this categorization, and all are distinct from pleasure. The emotion of interest has been distinguished from pleasure on the basis of cognitive appraisal processes—interest is positively associated with complex stimuli, but pleasure is negatively related to complexity (Turner and Silvia, 2006). Two other states that are distinct from pleasure and involve epistemic goals are *awe* and *beauty*. The emotion of awe is felt as one tries to accommodate vast novelty, the success of which leads to a powerful emotional state (Shiota et al., 2007). Awe can be and is frequently experienced as a negative and fear-like state when accommodation is unsuccessful. Beauty is defined as “the exhilarating feeling that something complex, perhaps to the point of being profound, might yield an understanding” (Armstrong and Detweiler-Bedell, 2008, p. 312). Beauty is distinguished from the pleasant on the basis of effort: pleasure is associated with fluent processing (Reber, 2012), but beauty relies on effortful processing that drives arousal and results in an exhilarating experience. Therefore, several aesthetic states are distinguished from simple pleasure. All are elicited by complex and novel situations where understanding is required but is effortful. Pleasure, on the other hand, is facilitated by ease of understanding.

From the individual differences perspective, two studies have distinguished pleasure and other aesthetic experiences through factor analysis techniques. Eysenck (1941) attempted to explain the presence of two factors in aesthetic preference. The first factor was easily attributable to valance, while the second was generally associated with preferences for the abstract. A core feature of abstract art is novelty and complexity, suggesting interest driven rather than pleasure driven preferences. More recently, Marković (2010) found that two factors describe aesthetic appreciation. These factors were labeled affective tone and aesthetic experiences. Descriptors “lovely” and “charming” loaded highest on affective tone, while aesthetic experience was associated with adjectives such as “exceptional” and “profound.” Thus, converging evidence and theory suggest that some aesthetic experiences are distinct from mild positive states of pleasure and that at the core of these states is the resolution of novelty and complexity, rather than fluent processing associated with pleasure.

Aesthetic states, like other emotions, are generated by appraisal process patterns (Ellsworth and Scherer, 2003). Interest occurs when a stimulus is appraised as novel yet understandable (Silvia, 2005). Novelty orientates and highjacks our attention, while the resolution of the novelty toward understanding leads to the positive experience of interest. This appraisal structure has been supported in response to art, poetry, and film (Silvia, 2005, 2008; Silvia et al., 2009a; Silvia and Berg, 2011). Pleasure and confusion are also predicted by the same appraisals but in different ways. Confusion is associated with appraisals of novelty and lack of understanding (Silvia, 2010). Pleasure is elicited by appraised understanding and negatively related to novelty (Turner and Silvia, 2006). The appraisal approach is therefore particularly useful in distinguishing differing aesthetic emotions and studying the underlying processes that facilitate them.

### Between Aesthetic Emotions and Aesthetic People

Appraisal theories of emotions have been used to further understanding of processes that underlie personality traits associated with emotional experiences. There are two ways in which personality is involved in the appraisal-emotion system: (1) appraisal strength—the tendency to appraise situations in a particular way—varies as a function of personality; and (2) appraisal-emotion relationships vary as a function of personality (Kuppens, 2009; Kuppens and Tong, 2010).

Openness/Intellect has been implicated in both of the aforementioned ways. Curiosity—a trait associated with Openness/Intellect (Mussel, 2010)—is associated with greater appraised understanding, which fully mediates the curiosity-interest relationship (Silvia, 2008). That is, curious people feel greater interest because they are better able to understand epistemic situations, which in turn predicts greater interest. This finding is consistent with the theoretical framework proposed by Mussel (2013) for Intellect traits. Within this framework, Intellect traits are associated with processes of seeking and conquering intellectually stimulating events, which map onto interest and understanding.

Further, within the experience of interest, novelty and understanding have been found to form two clusters with Openness/Intellect predicting membership in only one (Silvia et al., 2009a). Openness/Intellect was associated with the cluster in which novelty was a much stronger predictor of interest while understanding was less important, compared to the other cluster. This suggests that Openness/Intellect may moderate the interest-appraisal relationships predisposing those higher on Openness/Intellect to be more sensitive to novelty and less sensitive to understanding appraisals. One study has looked at the unique influence of the Openness and Intellect aspects on the processes and appraisal structure of interest in response to quotations. Openness was related to greater interest overall and a lessened reliance on understanding, while Intellect related to greater understanding (Fayn et al., 2015). This suggests that Openness and Intellect may relate to interest in different ways and that appraisal processes are useful for explaining these differences.

The influence of Openness/Intellect on the appraisal structure of pleasure and confusion, and the distinct influence of Openness and Intellect on the appraisal structure of interest, have not previously been tested. Taken together, previous findings indicate that appraisals can explain the mechanisms that underlie Openness/Intellect and its relationship with interest. Therefore, we aimed to evaluate the underlying processes associated with the Openness and Intellect aspects in order to understand whether those higher in either aspect are more aesthetically engaged and how the aspects manifest differently in aesthetic experiences.

### The Present Research

In summary, positive aesthetic experience is broader than liking and may be divided into two families of experiences: pleasure and the knowledge emotions. Openness/Intellect may influence both these states and the processes that underlie these traits. Therefore, we moved away from the predominant practice of evaluating *liking* artworks, in lieu of measuring distinct emotional states that have previously been implicated in the aesthetic experience. Additionally, by studying variability in appraisal-emotion relationships across multiple stimuli we were able to evaluate the way personality manifests in aesthetic experiences. Thus, the aims of the current research are to explore the relationship between Openness/Intellect and aesthetic appreciation by: (1) extending the states studied within personality-aesthetics relationships to pleasure, interest, and confusion; (2) evaluating the unique influences of the Openness and Intellect aspects; and (3) testing whether the Openness and Intellect aspects moderate the within-person appraisal processes that underlie these aesthetic emotional states.

Study 1 evaluated the differential influence of Openness and Intellect on different aesthetic states in response to visual art. In Study 2 we tested whether the appraisal processes associated with interest, pleasure and confusion can explain the relationships between Openness/Intellect and aesthetic appreciation, and whether the Openness and Intellect influence appraisal processes.

## Study 1

The purpose of this study was to test whether Openness and Intellect differentially predict states of interest, pleasure, and arousal. Based on past work on Openness and Intellect, we predicted that Openness would be a stronger predictor of aesthetic experience than Intellect.

### Method

#### Ethics Statement

This study was approved by the Human Ethics Committee of the University of Sydney. Written consent was obtained from all the participants before the experiment according to the established guidelines of the committee.

#### Participants

A total of 53 psychology students (74% female) participated in the study for course credit. Participants were aged between 17 and 42 years (*M* = 19.15 years, *SD* = 3.01 years). All participants were proficient in English ensuring comprehension of instructions.

#### Procedure

The study was conducted on computers over two 1-h sessions to minimize the influence of a long session of psychometric assessments on aesthetic appreciation. In the first session participants completed the Openness and Intellect scales, as well as other individual difference measures not relevant to the current study. In the second session—at least 1 h apart from the first—participants reported their thoughts and feelings in response to seven color images taken from published art books. The images were all in color and could broadly be described as modern art, comprising of both abstract and representational examples. The artists were: Dorosheva, Kadel, Kiefer, Magritte, Moki, Pollock, and Ryden.

#### Measures

##### Openness and Intellect

Openness and Intellect were assessed using the Big Five Aspect Scales (DeYoung et al., 2007). Each scale included 10 Likert style items on a five-point Likert scale (1 = *strongly disagree*, 5 = *strongly agree*) such as “I enjoy the beauty of nature” (Openness) and “I like to solve complex problems” (Intellect). The Openness scale is made up of items that reflect the Openness to Aesthetics, Feelings and Fantasy scales, while Intellect items include self-reported ability and Openness to Ideas items. The scale yields a full-scale Openness/Intellect score along with scores for the Openness and Intellect aspects. The internal consistencies for Openness (α = 0.86) and Intellect (α = 0.79) were good within the current sample.

##### Ratings of interest, pleasure, and arousal

After viewing each picture, people rated it on a series of seven-point semantic differential scales. The scales assessed feelings of *interest* (interesting-uninteresting, engaging-boring), *pleasure* (pleasing-displeasing, enjoyable-unenjoyable), and *arousal* (calm-aroused, sluggish-excited). Most of the items have been used in past research in research on emotions (e.g., Day, 1967, 1968; Silvia, 2005; Turner and Silvia, 2006). The items were reverse-scored and averaged; high scores indicate high levels of interest, pleasure, and arousal.

### Results and Discussion

The analyses were conducted with Mplus 7.2 (Muthén and Muthén, Muthén and Muthén) using maximum likelihood with robust standard errors. For interpreting effect sizes, we use the common guidelines (Cumming, 2012) of *r* = 0.10/0.30/0.50 as small/medium/large. **Table 1** reports descriptive statistics and correlations for the measures of personality and aesthetic experience.

**Table 1:** Descriptive statistics and Pearson correlations of personality variables with between-person aggregated ratings.

	*M*	*SD*	1	2	3	4	5
(1) Intellect	35.08	6.43	1	0.27	0.13	0.18	0.10
(2) Openness	40.23	5.39		1	0.28	0.34	0.39
(3) Interest	5.83	0.67			1	0.84	0.50
(4) Pleasure	5.52	0.74				1	0.47
(5) Arousal	4.67	0.72					1

*n = 53. All relationships above 0.18 are significant at 0.05 level, all those above 0.38 are significant at the 0.01 level, and all those above 0.49 are significant at the 0.001 level.*

**FIGURE 1:**
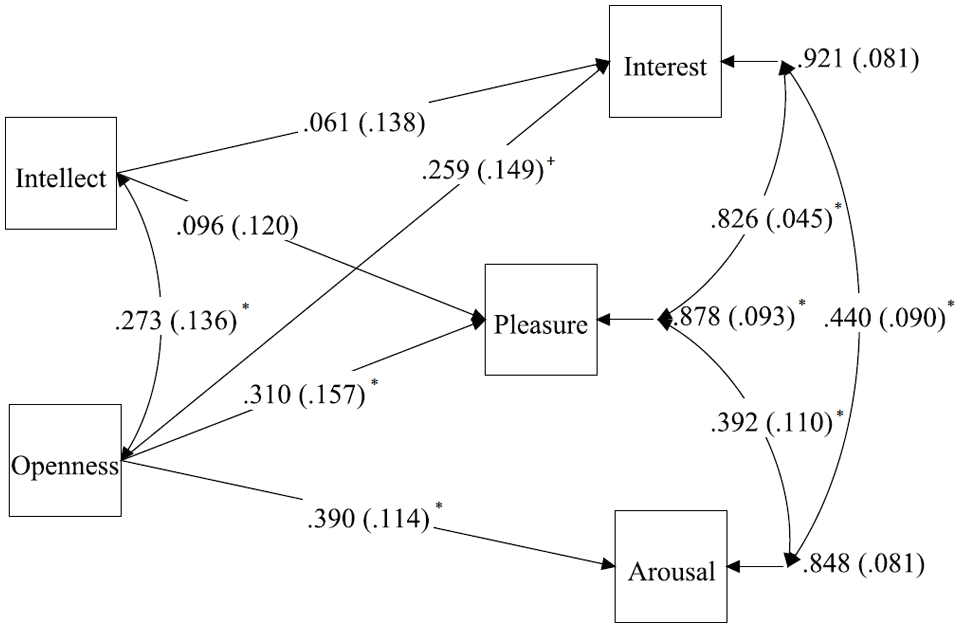
**Effects of Openness and Intellect on ratings of interest, pleasure, and arousal: Study 1.** *n* = 53. Note that the effect of Intellect on Arousal is β < 0.01 and hence not drawn. Standard errors are reported in brackets. ^∗^*p* < 0.05.

The zero-order correlations suggest, as expected, that Openness was associated with stronger aesthetic engagement than Intellect: Openness had stronger relationships, medium in size, with all three outcomes. To examine their differences more formally, we conducted a multivariate regression model in which Openness and Intellect were the two predictors and interest, pleasure, and arousal were the outcomes. **Figure 1** displays the model and results. The effects of Openness on interest, pleasure, and arousal were medium in size, and most were statistically significant; the effects of Intellect on interest, pleasure, and arousal, in contrast, were all near-zero or small in size. The results lend support to the utility of separating Openness and Intellect when evaluating individual differences in aesthetic states. Openness had notable relationships with the three types of aesthetic experience, whereas Intellect did not. Limitations of this study are the small sample size which we addressed in study 2, and a limited range on the Openness scale. Both of these limitations have a bearing on the strength of the results found in this study. Small sample sizes are an indication of underpowered studies, while range restrictions usually underestimate effect sizes.

## Study 2

Study 2 sought to extend these findings in several important ways. First, we shifted the range of emotional states that we assessed by focusing on *interest, pleasure*, and *confusion*. Whereas interest and pleasure have a long history in aesthetics research, confusion has only recently attracted attention among emotion researchers as a response to events that are unfamiliar and hard to understand (Silvia, 2010).

Second, to understand the processes underlying the Openness/Intellect-emotion relationships, appraisal processes were evaluated. The inclusion of appraisal processes can help determine why those higher in Openness/Intellect are more aesthetically sensitive—whether they are more or less emotionally responsive to appraisals. That is, we seek to determine whether Openness/Intellect can explain individual differences in appraisal-emotion relationships. As previously mentioned, Openness/Intellect moderates the appraisal structure of interest and relates to greater appraisals of understanding (Silvia, 2008; Silvia et al., 2009a). The current study extends this finding in several ways. First, we examine the two aspects of Openness/Intellect for their unique influence on aesthetic experience. Second, we test whether Openness and Intellect similarly moderate the appraisal structure of pleasure and confusion. We expect, as in Study 1, that Openness but not Intellect will be the aesthetically relevant aspect. Third, we included an additional individual difference measure to help clarify the roles of Openness and Intellect. A possible explanation for the relationship between Openness/Intellect and aesthetic appreciation is that those higher in Openness/Intellect have greater knowledge of the arts (Silvia, 2007a), which in turn predicts interest in art (Silvia, 2006). Art expertise has been shown to moderate the interest-appraisal relationships—experts are less reliant on understanding and more sensitive to novelty (Silvia, 2013)—a finding also associated with Openness/Intellect (Silvia et al., 2009a). This may indicate that the effects of Openness/Intellect on aesthetic appreciation are a function of expertise in the arts rather than a differences in personality. These variables have not been studied together in the context of aesthetic appreciation, therefore, we controlled for art expertise in the current study.

### Method

#### Ethics Statement

This study was approved by the Human Ethics Committees of the University of Sydney and the University of North Carolina at Greensboro. Written consent was obtained from all the participants before the experiment according to the established guidelines of the committees.

#### Participants

A total of 225 students from various degrees and majors (69% female) participated in the study for either course credit or $10 USD compensation. The students majors were 25.3% Physical Sciences, 21.8% Arts, 14.7% Psychology, 12% Health Sciences, 10% Business/Economics, 6.7% Social Sciences, 4.4% were undecided, and 4.9% had majors that did not fit into the categories presented as they were mixtures of more than one category. Participants’ age was between 18 and 56 years (*M* = 20.56 years, *SD* = 4.91 years). All participants were proficient in English ensuring comprehension of instructions.

#### Procedure

The data were collected during a 1-h session in groups ranging from 1 to 8 participants at a time. The study involved completion of self-report personality scales and ratings of 18 visual art images. We sought to include a broad scope of pieces ranging from traditional to contemporary art. The images were all in color and included both abstract and representational works. The artists were: Bacon, Blake, Goya, Hayuk, Kato, Kiefer, Magritte, Marc, Monroe, Pollock, Repin, Ryden, Schiele, Siqueiros, and Turner. The self-report scales came before and after the visual art ratings to avoid fatigue. All data was collected using Medialab (Jarvis, 2004) on computers. Images were presented in a random order, as were questions relating to the images; both controlled by the randomization algorithm within Medialab.

#### Measures

##### Openness and Intellect

As in Study 1, Openness and Intellect were assessed using the Big Five Aspect Scales (DeYoung et al., 2007). Each scale has 10 items on a five-point Likert scale (1 = *strongly disagree*, 5 = *strongly agree*).

##### Art expertise

Art expertise was measured using the *aesthetic fluency scale* (Smith and Smith, 2006), which assesses expertise by asking people how familiar they are with different figures and ideas from art history. The scale got participants to report their familiarity in response to 10 people and concepts (*Mary Cassatt, Isamu Noguchi, John Singer Sargent, Alessandro Boticelli, Gian Lorenzo Bernini, Fauvism, Egyptian Funerary Stelae, Impressionism, Chinese Scrolls, Abstract Expressionism*). The scale ranged from 0 (*I have never heard of this artist or term*) to 4 (*I can talk intelligently about this artist or idea in art*). It should be noted that the fluency scale assesses self-reported expertise in the arts and may be subject to overclaiming. However, the aesthetic fluency scale has been used widely used to assess expertise and has displayed good internal and external validity (e.g., Silvia, 2007a; Silvia and Barona, 2009; DeWall et al., 2011; Silvia and Nusbaum, 2011; Smith, 2014).

##### Emotions and cognitions in response to visual art

Participants viewed 18 images of various valance and style taken from various art books, previous studies, and the google images database. Participants could observe the image for as long as they wanted, but for a minimum of 5 s. A smaller version of the image was also visible while reporting on their thoughts and feelings.

For each image participants completed items assessing various emotions and cognitions. For emotional evaluations participants were asked: “Did you find this picture…” followed by items for *interesting, pleasing*, and *confusing*. Appraisal processes of novelty (*complex-simple, unusual-common*) and understanding (*hard to understand-easy to understand, comprehensible-incomprehensible*) were assessed using seven-point semantic differential scales. All scales had been previously used in assessments of aesthetic states (Silvia, 2005, 2010, 2013). In addition to the emotion items, we asked some behavior-like preference items, which are common in aesthetics research (e.g., Cooper and Silvia, 2009). For each image, participants were asked *I would like more information on this image, On Facebook I would “like” this image, On Facebook I would share this image on my wall*, and *I would like to own a copy of this*. Each item was answered with a binary NO/YES scale. The time taken to view each image was also recorded to evaluate whether Openness or Intellect were associated with longer viewing times.

### Results and Discussion

#### Data Reduction and Analysis

The items for the personality and aesthetic fluency scales were averaged to form overall scores. Internal consistencies for the BFAS Openness and Intellect scales, and the aesthetic fluency scale were good (see **Table 2**).

**Table 2:** Descriptive statistics and correlations between personality traits, aesthetic fluency and emotions.

	*N*	*M*	*SD*	1	2	3	4	5	6
(1) Openness	225	39.16	5.59	(0.76)	0.39	0.53	0.39	0.56	-0.28
(2) Intellect	225	36.23	5.51		(0.80)	0.39	0.11	0.27	-0.28
(3) Aesthetic Fluency	224	22.21	7.41			(0.83)	0.36	0.52	-0.26
(4) Interest	224	5.21	0.84				1	0.67	0.06
(5) Pleasure	224	3.51	0.83				0.52	1	-0.13
(6) Confusion	224	3.98	0.80				0.02	-0.20	1

*All relationships above 0.13 are significant at 0.001 level, and all those below 0.13 are not significant; correlation below the diagonal are within-person relationships; Cronbach’s alphas in parentheses.*

The large number of images viewed by each person allowed us to use multilevel models, which can estimate between-person effects, within-person effects, and their interactions (Silvia, 2007b; Nezlek, 2011). For the multilevel models, between-person predictors (Openness, Intellect, and Aesthetic Fluency) were centered at the sample’s grand mean and were rescaled by dividing the full scale score by the number of items in the scale. Within-person predictors (appraisals of novelty-complexity and understanding) were centered at each person’s own mean (Enders and Tofighi, 2007). The null model was used to evaluate intraclass correlation coefficients (ICCs). The ICCs indicated a significant amount of variance for interest (19%), pleasure (11%), and confusion (13%) at the between-person level.

**FIGURE 2:**
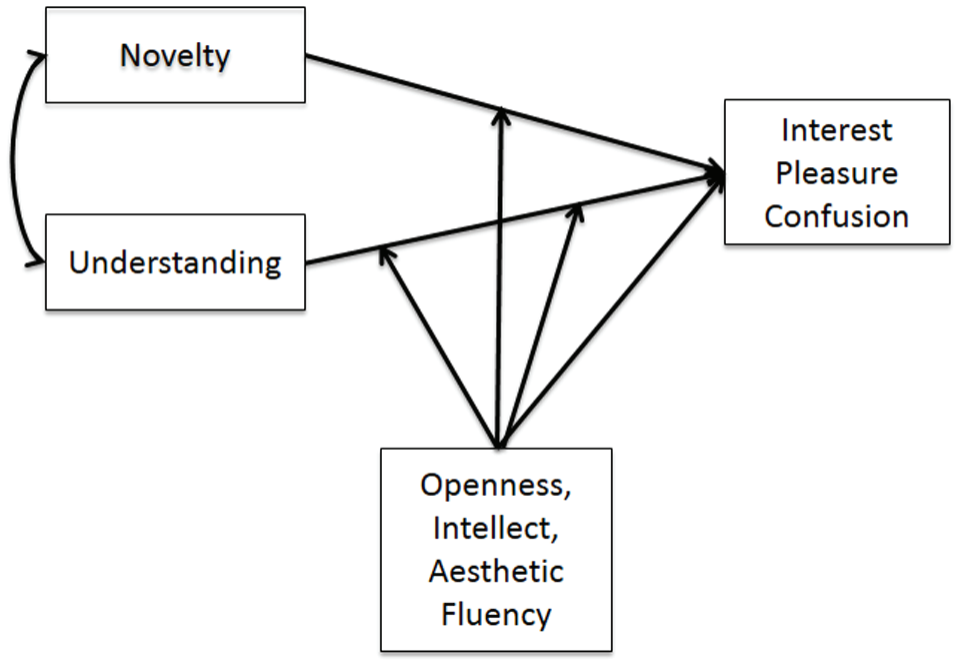
**A depiction of the multilevel models**.

The random slope and intercept models were tested separately for each emotion and are graphically depicted in **Figure 2**. The analyses were conducted with Mplus 7.2, using maximum likelihood with robust standard errors. All coefficients are unstandardized regression weights; some, where noted, are logistic effects. Estimation of power is a contentious topic within multilevel modeling due to the complexity of the parameters being estimated (Nezlek, 2011); by most standards the number of level 1 and level 2 units of measurement in our sample is sufficient to assume accurate estimations of the parameters of interest (Maas and Hox, 2005).

#### Descriptive Statistics and Bivariate Relationships

Openness and Intellect were both related to greater Aesthetic Fluency, pleasure, and lower confusion. Openness, but not Intellect, was related to greater interest (**Table 2**). The states of interest and pleasure had a strong overlap at the between and within person levels, and were unrelated to confusion at the between person level. Pleasure and interest differed from each other in their within-person relationship with confusion, interest was independent of confusion, but pleasure had a small negative relationship with confusion.

#### Overall Between-person Effects of Openness and Intellect on Emotions and Preference Ratings

Our first models examined the overall effects of Openness and Intellect on emotion ratings (interest, pleasure, and confusion) and on preference ratings (e.g., whether people indicated wanting to own a copy of the image). As expected, Openness and Intellect showed diverging relationships with these outcomes. Openness predicted finding the images significantly more interesting (*b* = 0.61, *SE* = 0.11, *p* < 0.001), more pleasing (*b* = 0.77, *SE* = 0.09, *p* < 0.001), and less confusing (*b* = -0.31, *SE* = 0.10, *p* = 0.003). Intellect, in contrast, predicted finding the images less confusing (*b* = -0.29, *SE* = 0.11, *p* = 0.008), but it didn’t significantly predict either interest (*b* = -0.06, *SE* = 0.10, *p* = 0.573) or pleasure (*b* = 0.09, *SE* = 0.09, *p* = 0.287).

For the preference ratings, a logistic model found that Openness significantly predicted the likelihood of wanting more information about the image (*b* = 1.65, *SE* = 0.32, *p* < 0.001), the likelihood of liking (*b* = 0.93, *SE* = 0.16, *p* < 0.001) and sharing (*b* = 1.09, *SE* = 0.25, *p* < 0.001) the image on Facebook, and the likelihood of wanting to own it (*b* = 1.14, *SE* = 0.18, *p* < 0.001). Intellect, in contrast, did not significantly predict wanting to learn more (*b* = -0.49, *SE* = 0.30, *p* = 0.101), liking (*b* = -0.08, *SE* = 0.16, *p* = 0.619) or sharing (*b* = 0.14, *SE* = 0.18, *p* = 0.402) the image on Facebook, or wanting to own it (*b* = 0.02, *SE* = 0.22, *p* = 0.942).

For view times—averaged across all stimuli—a regression model found that Openness significantly predicted greater viewing times (*b* = 206.29, *SE* = 62.85, *p* = 0.001). Intellect did not predict variance in view times (*b* = -0.39.06, *SE* = 63.89, *p* = 0.542).

In short, Openness and Intellect diverged in their relationships with aesthetic experience, preference ratings, and viewing times. Openness significantly predicted all of them, but Intellect predicted only feeling less confused.

#### Overall Within-person Effects of Appraisals on Emotions

The results for all multilevel models are presented in **Table 3**. These models evaluated the within-person main effects of appraisals on emotions. As in past work, interest was significantly predicted by appraisals of high novelty and high comprehensibility, and confusion was predicted by high novelty and low comprehensibility. Pleasure, in contrast, was more weakly predicted by novelty but predicted by comprehensibility, consistent with models that emphasize ease of understanding (Reber, 2012) and achieving insight and knowledge (Leder et al., 2012) as a source of liking.

**Table 3:** Multilevel models of within and between person predictors of aesthetic experiences.

Within-person predictors									
	Interest (DV)			Pleasure (DV)			Confusion (DV)		
Novelty	0.39^∗∗∗^ (0.03)			0.08^∗∗^ (0.03)			0.20^∗∗∗^ (0.02)		
Understanding	0.28^∗∗∗^ (0.02)			0.28^∗∗∗^ (0.03)			-0.56^∗∗∗^ (0.03)		
**Between-person predictors**									
		**Slopes**			**Slopes**			**Slopes**	
	**Intercept**	**N**	**U**	**Intercept**	**N**	**U**	**Intercept**	**N**	**U**

**Models 1–3**									
Openness	0.61^∗∗∗^ (0.11)	0.12^∗∗^ (0.04)	-0.06 (0.05)	0.77^∗∗∗^ (0.10)	0.16^∗∗^ (0.05)	0.02 (0.05)	-0.31^∗∗^ (0.10)	-0.01 (0.04)	-0.03 (0.04)
Intellect	-0.06 (0.10)	0.12^∗^ (0.05)	0.05 (0.04)	0.09 (0.09)	0.07 (0.06)	-0.04 (0.05)	-0.29^∗∗^ (0.11)	-0.06 (0.04)	0.01 (0.05)

**Models 4–6**									
Openness	0.45^∗∗∗^ (0.12)	0.11^∗^ (0.05)	-0.04 (0.05)	0.57^∗∗∗^ (0.09)	0.13^∗^ (0.06)	0.03 (0.05)	-0.23^∗^ (0.15)	0.03 (0.04)	<0.01 (0.05)
Intellect	-0.09 (0.10)	0.11^∗^ (0.05)	0.07 (0.04)	-0.01 (0.09)	0.05 (0.06)	-0.03 (0.04)	-0.27^∗^ (0.11)	-0.04 (0.04)	0.03 (0.05)
Aesthetic fluency	0.28^∗∗^ (0.08)	0.02 (0.04)	-0.05 (0.03)	0.34^∗∗∗^ (0.07)	0.04 (0.04)	-0.03 (0.04)	-0.13 (0.09)	-0.06^∗^ (0.03)	-0.06 (0.04)

*^∗^*p* < 0.05; ^∗∗^*p* < 0.01; ^∗∗∗^*p* < 0.001; N = Novelty-Interest slope; U = Understanding-Interest slope; Standard errors are reported in brackets.*

#### Personality as Predictors of Emotion Intercepts and Moderators of Appraisal-emotion Relationships

Openness and Intellect had different main effects on aesthetic experience, but do they moderate how appraisals influence aesthetic experience? These models included Openness and Intellect as between-person predictors of emotions and appraisal-emotion slopes. If a between-person trait significantly predicts a slope, then the relationship between an appraisal and an emotion shifts across levels of the trait. Prediction of intercepts implies that the overall mean of the emotion shifts according to trait regardless of appraisals. Both intercepts and slopes were modeled as random in these models.

Openness predicted larger intercepts for interest, pleasure, and smaller intercepts for confusion. Intellect predicted lower intercepts for confusion, but was not significantly related to interest and pleasure intercepts.

For *interest* (Model 1), the effect of novelty was moderated by both Openness and Intellect. For people high in Openness and Intellect, novelty was more strongly coupled to interest. No significant moderation effects appeared for understanding. For *pleasure* (Model 2), the effect of novelty was moderated by Openness but not Intellect. For people high in Openness, novelty was more strongly linked to pleasure. Follow up analysis on the difference between the novelty-pleasure slopes for Openness and Intellect indicated that they were not significantly different from each other (Wald test = 1.00, *df* = 1, *p* = 0.32). No significant moderation effects appeared for understanding. And for *confusion* (Model 3), in contrast, neither Openness nor Intellect moderated either appraisal. Neither the effect of novelty nor the effect of understanding on confusion varied across levels of Openness and Intellect.

Considered together, these results suggest that both Openness and Intellect are associated with greater sensitivity to novelty in the experience of interest, but only the Openness aspect is associated with greater sensitivity to novelty in the experience of pleasure. While the slope moderations by Openness and Intellect were not found to differ from each other, the moderating influence of Openness was significant, while the influence of Intellect was not. Finally, Openness, but not Intellect, was associated with greater pleasure and interest overall.

#### Exploring Art Expertise

Our final models explored the roles of art expertise (measured with the aesthetic fluency scale). To examine art expertise, we included it alongside Openness and Intellect to see if it reduced their effects. As we discussed earlier, such a result would suggest that the effects of personality are largely carried by acquired expertise about the arts.

The inclusion of art expertise didn’t change any of the Openness and Intellect findings with respect to interest, confusion and pleasure. This suggests that the effects of Openness and Intellect are not driven by greater expertise in the arts. For *interest* (Model 4), neither the effect of novelty nor the effect of understanding was moderated by art expertise, but expertise was related to greater intercepts in the model. For *pleasure* (Model 5), neither the effect of novelty nor the effect of understanding was moderated by art expertise, but expertise was related to greater intercepts in the model. And for *confusion* (Model 6), art expertise moderated the effect of novelty, but not understanding; in contrast, neither Openness nor Intellect moderated either appraisal. This suggests that novelty is less related to confusion for those with greater art expertise. These results suggest that the novelty-interest and novelty-pleasure moderation are not influenced by art expertise but are rather driven by Openness.

## General Discussion

Openness/Intellect is the personality domain that best explains individual differences in aesthetic appreciation. However, the research linking actual art appreciation to the domain has several issues. First, as discussed in the introduction the focus on *liking* artworks is limited, as aesthetic experience is much broader and richer than mild feelings of pleasure (Silvia, 2009). Second, there’s a risk of circularity in the relationship, given that items about aesthetic engagement appear on all major Openness to Experience scales. Without examining why this relationship exists, not much is added to our understanding of Openness/Intellect and aesthetics. In this research, we broadened the range of aesthetic emotions and examined appraisal mechanisms that could explain differences in aesthetic experience as a function of Openness/Intellect. Art expertise was evaluated alongside personality to test whether the influence of Openness and Intellect on aesthetic appreciation can be explained by greater art knowledge.

As predicted, Openness/Intellect reflected individual differences in aesthetic experiences—both pleasure and the knowledge emotions. The strength of the relationship was particularly driven by Openness as opposed to Intellect, supporting the distinction in the aspects based on perceptual versus abstract engagement (DeYoung, 2014). Mechanisms for these relationships were also discovered through differences in appraisal-emotion relationships. The Openness/Intellect aspects predicted reactivity to novelty appraisals in experiences of interest. While the novelty seeking core of Openness/Intellect has previously been suggested (Woo et al., 2014), our study provides within-person process evidence for this special relationship with novelty and demonstrates that those higher in Openness/Intellect are *reactive* to novelty in their experiences with interest. Openness diverged from Intellect in the experience of pleasure. Intellect did not predict individual differences in the processes associated with pleasure, but novelty was a stronger predictor of pleasure for people high in Openness. Further, Openness predicted greater interest and pleasure regardless of how artworks were appraised, further distinguishing it from Intellect. Openness and Intellect were related to lower levels of confusion, but variance in appraisal-emotion relationships was not associated with either aspect.

Finally, the possible confound of art expertise was evaluated as an explanation for the Openness-aesthetic emotions relationship. The inclusion of art expertise did not influence any of the Openness-aesthetic emotion relationships, suggesting that the effects were particular to the personality variables rather than greater expertise. Expertise did predict greater interest and less confusion overall, and it was related to a smaller relationship between novelty and confusion.

Together these findings provide an important update for our understanding of the relationship between the Openness/Intellect and aesthetic emotions. Particularly, our findings show that Openness, as opposed to Intellect, is the aspect of the aesthetically engaged, and provide a process based understanding for w*hy* those higher in Openness are more aesthetically engaged. Finally, methodological differences between this and previous research on personality and aesthetics highlight the advantages of the current approach.

Within this paper we assume rather that test a causal flow from personality to emotion states. That is, we assume that personality reflects biologically driven consistencies in emotions, cognitions, and behavior. Therefore, personality is treated as an antecedent of states. Similarly, appraisals are considered to be antecedents of emotions. For interest, both appraisals, when experimentally manipulated, have been shown to influence interest (Silvia, 2005). Thus, within this paper, we treat appraisals as causing emotions.

### Advantages of the Current Method

There are two methodological differences between the current method and most of the research on personality and aesthetics. First, we moved away from the predominant practice of evaluating *liking* artworks and shifted toward measuring distinct emotional states that have previously been implicated in the aesthetic experience. Liking is a common and important aesthetic response—mild feelings of pleasure might be the most common everyday aesthetic experience—but it is only one of many important experiences people have in response to the arts (Silvia, 2009). Second, we explored both within- and between-person effects. The integration of dispositional and situational variables has long been advocated (Cronbach, 1957; Underwood, 1975), but it is uncommon for aesthetics research to examine effects at the within-person level of analysis, which is the natural level for examining how appraisals influence emotional responses (see Silvia, 2007b; Nezlek, 2011).

### The How and Why of Openness/Intellect and Esthetics

Previous research has demonstrated that Openness/Intellect is related to differences in appraisal processes for the emotion of interest (Silvia, 2008; Silvia et al., 2009a). The current research builds on these findings in two important ways by: (a) evaluating the independent roles of Openness and Intellect in interest-appraisal processes; and (b) evaluating differences in pleasure-appraisal and confusion-appraisal processes.

Openness and Intellect were both associated with reactivity to novelty in the experience of interest suggesting that novelty sensitivity is at the core of the domain. However, Intellect, as opposed to Openness, did not reflect greater interest overall. This suggests that being higher on Intellect is reflective of lower than average levels of interest when novelty is not found in an artwork, yet higher than average interest for novel artworks. Conversely, Openness was related to greater interest regardless of appraised novelty suggesting that while novelty is preferred, greater interest is experienced even in the absence of it. The sensitivity to novelty in the experience of interest for both Openness and Intellect provides a possible process explanation for part of the Openness-Fluid-Crystallized-Intelligence (OFCI) model which proposes a developmental link between Openness/Intellect and fluid intelligence (Ziegler et al., 2012). Ziegler et al. (2012) propose that being open increases learning opportunities, thereby increasing fluid intelligence. Our findings suggest that Openness/Intellect is associated with a sensitivity, through interest, to stimuli and situations that are appraised as novel and complex. This preferential engagement with challenging information could support the pathway from Openness/Intellect to gains in fluid and crystallized intelligence.

While the Openness and Intellect aspects reflect quantitative differences in the appraisal structure of interest, qualitative differences are present in the experience of pleasure. Openness, but not Intellect, was associated with the presence or absence of a pleasure-novelty relationship. Studies have shown quantitative differences in appraisal structures—the appraisal structure remains constant yet the predictive *strength* of an appraisal varies as a function of a trait (Kuppens and Tong, 2010). However, few studies have found qualitative differences in appraisal structures. Our findings indicate that those higher in Openness experience pleasure as a function of novelty and understanding, while those lower on the aspect are only influenced by understanding. The idea that understandable things are pleasant is congruent with fluency based aesthetic theories where things that are easily understood are pleasant to the beholder (Reber, 2012). Our findings suggest that this may primarily be the case for people lower on Openness. For those higher on Openness, pleasure is also influenced by the novelty of an artwork.

This finding has important implications for aesthetic theories. Fluency based accounts are at odds with interest based accounts. Interest requires novelty, whereas fluency-based aesthetic experiences are a function of easy processing. This distinction maps nicely onto interest and pleasure. Interest is experienced in the face of novelty and pleasure is experienced when processing requires little effort. Our research suggests that individual differences both complicate and clarify this distinction. It seems that the influence of fluent processing in the experience of aesthetic pleasure is dependent on trait standing. Those higher in Openness are sensitive to novelty and complexity in their experience of pleasure. Conversely, pleasurable experiences for those lower on Openness are not predicted by stimulus novelty.

### Openness/Intellect Model

These findings add to the growing empirical consensus for the utility of studying Openness and Intellect as separate aspects of the broader domain. The distinction previously proposed—Openness as exploration through perception, and Intellect through learning and abstract information (DeYoung, 2014)—is supported with Openness reflecting greater pleasure and interest and less confusion in response to visual art. While Intellect was also found to play a role in the processes that facilitate interest, this role does not predict greater aesthetic reactions but rather reflects a preference for the novel, and a lesser tendency to feel confusion in response to visual art. The relationship between Intellect and interest in art, when controlling for Openness, is not evident at the between-person level, but is apparent when within-person processes are considered. Future studies are encouraged to explore the differential influence of Openness and Intellect on interest in non-perceptual stimuli such as science and philosophy to gain further insights into this useful separation of the Openness/Intellect domain.

## Conclusion

Henry James saw art as central to life and beauty, and this attitude, like that of many other creative people, was likely a function of his Openness. We aimed to extend our understanding of the role personality plays in common aesthetic experiences: pleasure, interest, and confusion. Our findings suggest that Openness, as opposed to Intellect, is the personality core of aesthetic experiences, and that the relationship persists because those higher in Openness are more sensitive to novelty in artworks and experience greater engagement overall, predisposing them to feel more interest and pleasure in response to the arts.

## Conflict of Interest Statement

The authors declare that the research was conducted in the absence of any commercial or financial relationships that could be construed as a potential conflict of interest.
